# Diffuse large B-cell lymphoma, not otherwise specified of the palate: A case report

**DOI:** 10.4317/jced.51127

**Published:** 2013-12-01

**Authors:** Giovanna R. Souto, Thaís SF. Pereira, Alexandre F. Castro, Ricardo A. Mesquita

**Affiliations:** 1 DDS. MSc. Department of Oral Surgery and Pathology.School of Dentistry. Universidade Federal de Minas Gerais; 2 DDS. Department of Oral Surgery and Pathology.School of Dentistry. Universidade Federal de Minas Gerais; 3 MD. Oncomed. Belo Horizonte. Minas Gerais, Brazil; 4 MD. Hospital Felício Rocho. Belo Horizonte. Minas Gerais, Brazil; 5 DDS. PhD. Department of Oral Surgery and Pathology.School of Dentistry. Universidade Federal de Minas Gerais

## Abstract

Diffuse large B-cell lymphoma (DLBCL) is the most frequent type of non-Hodgkin´s lymphoma found in oral and maxillofacial regions. A large number of cases may be biologically heterogeneous, which are commonly defined as DLBCL, not otherwise specified (NOS) by the World Health Organization (WHO-2008). The present case reports on an ulcer of raised and irregular edges, found on the border between the hard and soft palate, as the first and only manifestation of an extranodal non-Hodgkin lymphoma in an 85-year-old patient. Incisional biopsy was carried out, and the specimen revealed a proliferation of large lymphoid cells suggestive of diffuse large cell lymphoma. An immunohistochemical analysis was performed. EBV-RNA was assessed by in situ hybridization that also proved to be negative. Immunohistochemical and EBV analyses are important to avoid delays and inappropriate treatment strategies. Although advanced age is considered an adverse prognostic factor, early diagnosis did prove to be a key contributory factor in the cure of non-Hodgkin lymphoma.

** Key words:**Diffuse large B-cell lymphoma, elderly, EBV.

## Introduction

Diffuse large B-cell lymphoma (DLBCL) is the most frequent type of non-Hodgkin´s lymphoma (NHL) found in the maxillofacial region (1-4). DLBLC affects both the osseous and soft tissues, and favored sites include tonsils, palate, and parotid glands (3). Patients most commonly present a quick, painless swelling; in up to 40% of patients, an extranodal site can also be identified (5,6), including oral tissues (3). The incidence of DLBCL oral lesions increases with age, occurring, on average, in the 7th and 8th decades of life (2,3). The etiology remains unknown, and the immune deficiency is considered a risk factor (5). It is considered an aggressive but treatable neoplasm, with a variable clinical course (5,7).

DLBCL is a proliferation of large B lymphoid cells with nuclear sizes equal to or exceeding the macrophage nuclei or more than twice the size of a lymphocyte (8). Authors have subdivided DLBCL in distinct disease entities according to morphological, biological, and clinical features. Variability in response to treatment is often observed, emphasizing the diversity of this disease (5,9). The World Health Organization (WHO-2008) defines the cases in which there are no clear and accepted criteria for the subdivision of DLBCL, not otherwise specified (NOS) (8,10). In addition, a distinct subgroup of DLBCL was identified as Age-related Epstein–Barr virus (EBV) associated diseases that appear to occur most often at advanced ages, namely EBV-associated B cell lymphoproliferative disorders (11,12). Thus, the investigation of EBV infection is important in elderly patients that present DLBCL without the association of disease immunosuppression. In this light, this workaimed to present a case report of DLBCL-NOS of the palate oral cavity in an elderly patient, as well as to carry out a review of the relevant literature.

## Case report

**Figure 1:**
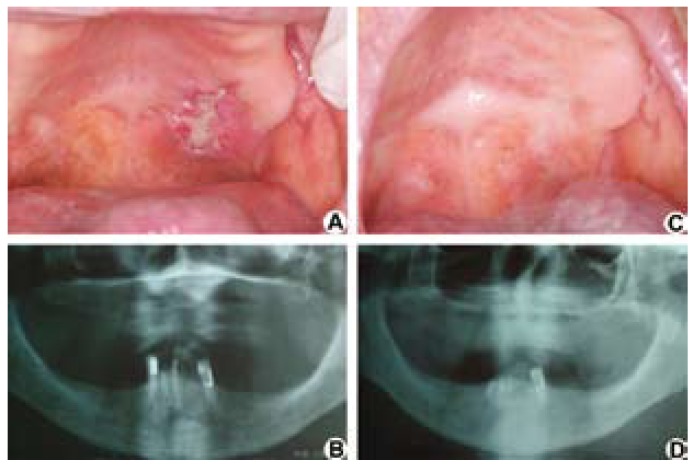
A) Initial clinical image. An ulcer of raised and irregular edges could be observed on the border of the hard and soft palates. B) Initial panoramic radiographshowed no bone changes. C) Clinical image and D) panoramic radiograph after thirty-one months of diagnosis and treatment, in which the lesion’s total clinical resolution could be observed.

**Figure 2:**
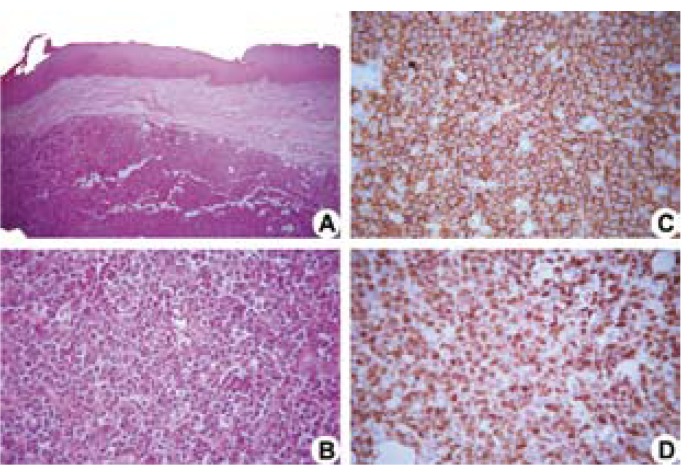
Histopathological sections stained with hematoxylin-eosin show a neoplastic proliferation of large lymphoid cells (A, 50x original magnification) and the presence of neoplastic cells with coarse chromatin and inconspicuous nucleoli (B, 400x original magnification). Immunopositive cells of anti-CD20 (C, 400x original magnification, Streptoavidin-biotin) and anti-Ki-67 (D, 400x original magnification, Streptoavidin-biotin).

An 85-year-old woman was referred to the Oral Medicine Clinic of the School of Dentistry at Universidade Federal de Minas Gerais (UFMG) for evaluation of a lesion on the palate, with a two-weekevolution and complaints of pain. Intraoral examination identified an ulcer of raised and irregular edges, measuring 30 mm at its largest diameter, covered by a yellowish-white membrane, located on the border of the hard and soft palate on the left side (Fig. 1). The patient was reportedly hypertensive, presented diastolic heart dysfunction, and had episodes of dizziness. Panoramic radiographsshowed no bone changes (Fig. 1). The differential clinical diagnoses included a malignant neoplasm of miscellaneous origin or oral metastasis. An incisional biopsy was carried out,and the H&E stained specimen revealed a proliferation of large lymphoid cells with a high nucleus:cytoplasm ratio, coarse chromatin, and inconspicuous nucleoli with abnormal mitotic figures (Fig. 2). The morphology was suggestive of diffuse large cell lymphoma and an immunohistochemical analysis was performed. Positive neoplastic cells for anti-CD20 (Fig. 2), CD79a, CD10, MUM-1, Bcl-6, and Bcl-2, as well as a labeling index of 90% for Ki-67 (Fig. 2), could be observed. Neoplastic cells were negative for anti-CD3, CD5, and CD138. The EBV RNA assessed by in situ hybridization using EBER oligonucleotides proved to be negative. Immunohistochemicalanalysis of the EBV-latent gene products using latent membrane protein-1 (LMP-1) also proved to be negative. The diagnosis was of DLBCL-NOS. The patient was then referred to a physician. Computerized tomography scans of the chest, abdomen, and pelvis, and an iliac crest bone biopsy showed no signs of lesions. Signs of the involvement of nodal sites were not observed. The employed chemotherapy treatment was that of the R-CHOP protocol, applied every 21 days, over a three-month period (Rituximab - 500 mg D1; Cyclophosphamide - 800mg D; Vincristine - 1.0 mg D1, and prednisone - 80g D1-D5). After the 4th cycle of chemotherapy, the patient was referred for radiotherapy. Radiotherapy treatment was 4320 cGy in lateral cervical facias, biopostos, and parallel fields, as well as the 4320 cGy indirect field under supraclavicular fossa, for 25 days. Total clinical resolution of the lesion after treatment could be observed. After follow-up carried out for thirty-one months after treatment, the patient can now be considered clinically and radiographically free of disease (Fig. 1).

## Discussion

In the present case report, the patient presented an ulcer of raised and irregular edges, on the border of the hard and soft palate, with a diagnosis of DLBCL-NOS. After the physician’s assessment, the mouth was considered the primary site of the disease, with no other lesions appearing in other regions. The patient, at the time of the biopsy, was 85 years of age. Although DLBCL can occur in a broad age range, the mean age most commonly occurs in the 7th and 8th decade of life (2,3,6). The differential diagnosis was performed by means of an incisional biopsy. DLBCL is often mistaken for squamous cell carcinoma and benign or malignant neoplasias of the palatal salivary glands. Immediate and accurate diagnostic procedures were performed, including immunohistochemical and EBV analyses to avoid delay and inappropriate treatment strategies.

The lymphomas are neoplasms that are classified in two groups: in Hodgkin’s lymphomas, by the presence of the Reed–Sternberg cells, and in non-Hodgkin’s lymphomas, by all other lymphoid neoplasms. For the NHL group, up to 40% are observed in an extranodal site, and 2-3% of these extranodal cases may arise primarily in the oral cavity and jaws. Studies of oral NHL demonstrated a major predominance of the DLBCL subtype, which accounts for 50% to 68% of all oral NHL (2,7).

In 2008, the International Agency for Research on Cancer (IARC) published the 4th edition of the WHO Classification of Tumors of Hematopoietic and Lymphoid Tissues (8,10). New subtypes of DLBCL were defined and cases that did not present sufficient criteria to classify them as distinct variants weredesignated as DLBCL-NOS. In addition, EBV-positive DLBCLs in the elderly have also been identified as separate entities. These are clinically aggressive and occur more often in extranodal rather than nodal sites. These lesions also appear to occur most often at an advanced age, occurringmostly in patients >50 years of age, with no known immunode?ciency or prior lymphoma. These are believed to be related to immunological deterioration derived from the aging process, likely due to decreased immune surveillance (11-14).Therefore, it is necessary to investigate EBV-infection in samples diagnosed as DLBCL and in patients over 50 years of age. However, in the present case, the search for the EBV-infection, using both methods ofin situ hybridization for EBER and immunohistochemistry for LMP1 protein, proved to be negative.

Immunohistochemical markers have been reported to be associated with prognostic effects for DLBCL (8). In the present case report, a high proliferation index by means of Ki-67 staining could be observed. Positive expression of anti-CD20 could also be observed. Moreover, the addition of the anti-CD20 antibody rituximab in the chemotherapy treatment considerably improved the survival rate of patients with DLBCL and proved to eliminate the negative impact of the Bcl-2 expression (15). Studies have suggested that positive expressions for Bcl-2 and MUM-1 were associated with poor prognosis (16-18). However, positive expression for markers such as anti-CD-10 and Bcl-6 can be associated with favorable prognoses (16). In the present case, positive expression for CD-10 and Bcl-6 could be observed. Nonetheless, other studies have suggested that immunohistochemical subtypes of DLBCL based on CD-10, MUM-1, and Bcl-6 have little clinical value (19).

In contrast, Chung et al. (20) suggest that expression patterns of the panel of

Germinal-center B-cell (GCB) markers, such as CD-10 and Bcl-6, and GCB activation markers, such as MUM-1 by immunohistochemistry can be correlated with the prognosis of patients with DLBCL. This study explained that the discrepancies regarding the prognostic significance of CD-10 or Bcl-6 expression in DLBCL reported in prior literature is related to the evaluation of these two markers (CD-10 and Bcl-6) individually without the simultaneous evaluation of activation markers, such as MUM-1. Bhattacharyya et al. (21), in a study with 13 primary oral DLBCLs, show that non-GCB lesions, when compared to GCB lesions, may exhibit a poorer prognosis. Although the small number of cases and short follow-up periods (less than 5 years) are a limitation of this study, the authors considered that the stratification of DLBCL by immunohistochemistry is possible and represents a relatively inexpensive method for the classification of DLBCL subtypes. In the present case, the DLBCL was classified as a subtype of a GCB lesion.

In addition to the prognostic factors, more unfavorable results could be observed in older patients, including a poor hematologic tolerance to chemotherapy, a higher risk for treatment-related toxicity due to altered drug metabolism, and a worse immune system. These factors lead to a less effective NHL therapy (22,23). Older patients with aggressive lymphoma present a worse outcome than do corresponding younger patients (23). Thus, age itself is considered an adverse prognostic factor. Gutierrez et al. (13) reported an increase in the incidence of DLBCL in the elderly. Multiple factors, including an increase in life expectancy, may well be an important factor contributing this increase. In this context, an immunological deterioration caused by the aging process may well explain the occurrence of EBV-positive DLBCL of the elderly in patients without predisposed immunological disorders (11,12).

## Conclusion

In the current case, although the patient was of an advanced age with the presence of hypertensive disease and diastolic heart dysfunction, he did undergo an excellent follow-up treatment. Although doses of R-CHOP had been adjusted for the age and heart condition of the patient, the early diagnosis did prove to be a key contributory factor to this outcome. The extent of tumors in the early-stages of DLBCL may well contribute to the choice of therapeutic strategy (24), which can have a direct impact on the survival of the patient.
